# Characterization of simulation centers and programs in Latin America according to the ASPIRE and SSH quality criteria

**DOI:** 10.1186/s41077-021-00188-8

**Published:** 2021-11-12

**Authors:** Soledad Armijo-Rivera, Felipe Machuca-Contreras, Norma Raul, Saionara Nunes de Oliveira, Ismael Ballesteros Mendoza, Héctor Shibao Miyasato, Diego Andrés Díaz-Guio

**Affiliations:** 1 Núcleo de Simulación Interdisciplinar, Facultad de Medicina Clínica Alemana de Santiago Universidad del Desarrollo, Avenida Las Condes 12438 Lo Barnechea, Santiago, Chile; 2 Universidad Autónoma de Chile, Pedro de Valdivia 425, Providencia, Santiago, Chile; 3 Clinical Simulation Training Center, Hospital de Alta Complejidad en Red El Cruce Dr. Néstor Carlos Kirchner, Ministry of Health, Buenos Aires, Argentina; 4 Department of Nursing, Federal Universidade Federal de Santa Catarina, Florianópolis, Brazil; 5 Simulation Center, Faculty of Medicine, Universidad Diego Portales, Ejército, 141 Santiago, Chile; 6 Simulation Center, Integrated School of Medicine, Nursing and Dentistry, Universidad Peruana Cayetano Heredia, Lima, Perú; 7 Education and Simulation Research Group, VitalCare Centro de Simulación Clínica, Faculty of Medicine Universidad Alexander von Humboldt, Armenia, Colombia

**Keywords:** Simulation training, High fidelity simulation training, Quality assurance, Health care education, Medical simulation centers

## Abstract

**Background:**

Latin American clinical simulation has had an important development; there are no studies that characterize simulation centers and programs in the entire region. The aims of this work are to characterize the current state of simulation-based education in the health sciences, to determine the structure of Latin American simulation centers in terms of teaching, research, and continuing medical education (CME), as well as to determine the perception of quality based on international standards of simulation practices for the directors of Latin American centers.

**Methods:**

A quantitative, descriptive, cross-sectional study with a demographic questionnaire and a Likert-type survey was conducted to the directors of the simulation centers found in Latin America.

**Results:**

Four hundred eight simulation centers were documented, the survey was answered by 240 directors, and the data from 149 were complete responses on the 42 quality self-perception scale and considered valid on further analyses related to the quality of the programs. Most of the centers that responded correspond to Chile, Brazil, and Mexico (37.5%, 18.1%, 12.7%). 84% of the centers are university-based, and 71% of the centers are medium-sized, with less than 10 instructors (54%). The directors are mostly women (61.7%), medical doctors (50%), and nurses (40%), with clinical specialization (37%), master’s degree (53%), and doctorate (13%). 75% have completed a simulation instructor course, and 6% have developed a fellowship. Most consider the maintenance of international quality standards to be relevant in their centers, mainly in reflective training techniques, ethical aspects, and adequate learning environments.

**Conclusions:**

Simulation-based education in health sciences has had an increasing development in Latin America, within a university environment, in an important academic specialization process that seeks to adhere to high-quality standards to improve training and development of clinical skills, human factors, and critical thinking. We recommend starting accreditation processes in Latin America and studies that measure the quality of simulation-based education in our region, based on objective observations more than in self-reporting.

## Background

Latin America is a region of the American continent whose languages, derived from Latin, are mainly Spanish and Portuguese. This territory is made up of approximately 20 nations and has an extension of 22,000,000 km^2^, where about 626 million people live, with ethnic, cultural [1], economic, and public education financing diversity [2].

Latin America is different from regions in which clinical simulation training and research criteria or recommendations are available for simulation-based education such as Europe, the UK, the USA, and Canada [3–5].

Simulation has been reported in Latin America as a teaching tool in prelicensure [6, 7], postgraduate [8], and cardiopulmonary resuscitation programs [9], and as an assessment tool inside OSCEs (Objective and Structured Clinical Examination) [10–12]. Information regarding simulation centers in Latin America is scarce [13, 14]. There are no studies that characterize simulation centers and their programs or quality.

The quality of clinical simulation occupies an important part of the agenda of scientific societies in Europe and North America, focusing both on the standards and recommendations of good practice [15], as well as on the accreditation criteria to measure quality. The accreditation criteria for the simulation centers of the Society for Simulation in Healthcare (SSH), which consider elements of systems integration [3], the criteria of the Association for Medical Education in Europe (AMEE) for accreditation of programs [4], the standards for simulation training developed by the International Nursing Association for Clinical Simulation and Learning (INACSL) [5], and the Association of Standardized Patient Educators (ASPE) [16] are examples of standards of quality that do not exists for Latin America.

Currently, in Latin America, there is the need to work on standardized aspects of the quality of simulation-based education; nonetheless, until now, there is no consensus instrument to measure the quality of our centers and programs.

The aims of this work are to characterize the current state of simulation-based education in the health sciences, to determine the structure of Latin American simulation centers in terms of teaching, research, and continuing medical education (CME), as well as to determine the perception of quality based on international standards of simulation practices for the directors of Latin American centers.

## Methods

### Study design

A quantitative, descriptive, cross-sectional study using an online self-report instrument was conducted.

### Setting and participants

For this study, we considered a simulation center or simulation program as an organization with dedicated resources, and a mission targeted to the use of simulation for education, assessment or research, that uses a substantial component of simulation as a technique [3].

Directors of simulation centers were asked to respond representing simulation centers in Latin American Spanish- and Portuguese-speaking countries.

An intentional sample was selected based on the defined population. A database was created with the contact emails that appeared on the institutional websites and was complemented by a snowball sampling technique to cover the greatest extent of the universe in the cases where a contact email was not available at the institutional website. To include centers that do not have a website, we used the information available from congress contacts. Once the database was constructed with the information known to the authors, we expanded it, identifying simulation center directors in each country from whom we requested contact information for centers not yet included in the database. No databases of country societies or the Latin American federation were used.

We also used data available on population, and the percentage of global Gross Domestic Product (GDP) spended on education and health in Latin American countries, obtained from public information on the CEPAL website for 2018 [17].

### Instrument development

A group of researchers (two nurses and five medical doctors) trained in simulation, with experience in administering simulation centers and research, belonging to the Federación Latinoamericana de Simulación Clínica (FLASIC, www.flasic.org), constituted a committee to design the research protocol to develop the instruments.

Based on a literature review that included the accreditation criteria of SSH and ASPIRE, surveys used to characterize worldwide simulation centers [18–20], centers in European [21, 22] and Latin American single countries [14], and some definitions to report simulation centers resources and activities [23]. A two-part bilingual instrument (Spanish and Portuguese) was designed [24].

The selection of the SSH criteria was based on the fact that they include systems integration criteria that we needed to characterize the simulation centers of clinical institutions, and the ASPIRE criteria because they are based on elements of Medical Education. In addition, both SSH and ASPIRE have center and program accreditation processes. The survey includes the main criteria of both SSH and ASPIRE.

The first part is a characterization questionnaire with 19 questions, focusing on center information (country, type of institutions, infrastructure, metrics of activities, human resources and directors profile, simulation resources) and program orientations. In relation to the size of the centers, they were classified into groups according to the square meters reported (small <122 m^2^, medium 122–1500m^2^, large 1500–2900m^2^, very large> 2900m^2^). For the metrics of activities used in the questionnaire (number of participants per year, hours per participant, number of activities, and number of hours of room use), an explanation of how to calculate them was included [23].

The second part was developed through a modified e-Delphi method based on the opinions of six Latin American experts to assess the self-perception of quality. The expert profile was a professional with more than 7 years of experience and postgraduate training in educational sciences and clinical simulation who had experience in administering simulation centers and training instructors.

A first draft of the second part of the instrument was created in Spanish with six dimensions and 42 items based on the accreditation criteria for SSH [3] and ASPIRE [4] simulation centers. This version passed through a three-step iterative creation process (e-Delphi) until it reached a complete consensus. The semantics, wording, and spelling were adjusted at the first Delphi step. The following stages did not generate changes in the instrument. As a result of that process, a preliminary Spanish version of this questionnaire was obtained [24]. The revised questionnaire maintained the initial number of dimensions and items.

The instrument was translated to Portuguese by a researcher, native in Portuguese and proficient in Spanish. A backward independent translation was performed from Portuguese to Spanish to corroborate the first. Finally, in terms of the semantics and cultural equivalence for the study, the Portuguese version was reviewed by two simulation instructors with Portuguese as their native language [26, 27].

The bilingual final instrument was composed of a demographic questionnaire with 19 items and a 42 items quality self-perception questionnaire Likert-type (1 = totally disagree to 5 = totally agree).

The average lickert per item and dimension was calculated. A score was also calculated for each dimension and for the total scale, adding the score for each item.

### Data collection

This study was carried out between January and May 2019. The survey was hosted on the Survey Monkey® platform (https://es.surveymonkey.com/) and sent by email to the directors of the simulation centers in Latin America, with monthly reminders (four email reminders). There were no incentives for participation or completion of the survey.

### Statistical analysis

The use of descriptive statistics for the characterization of the sample was considered. Cronbach’s alpha was calculated as a measure of internal consistency of the self-perception of the quality questionnaire (alpha value> 0.70) [25, 28]. In the 42 items self-perception questionnaire of the instrument, good internal consistency with Cronbach’s alpha of 0.95 was found.

The statistical package IBM Corp. Released 2015. IBM SPSS Statistics for Windows, Version 23.0. Armonk, NY: IBM Corp was used. The correlation analysis with demographic and economic data was performed with Microsoft Excell (version 16.52).

### Ethical approval

Participants informed consent were obtained. The ethical committee approved the Research design in the Universidad del Desarrollo de Chile (CEI 46/2018) and the Federal University of Santa Catarina of Brazil (Parecer do Comitê de Ética N° 3.206.561).

## Results

### Characteristics of Latin American simulation centers

Four hundred eight directors of simulation centers in Latin America were contacted. The distribution of those centers goes between 1 and 136 per country.

**Fig. 1:**
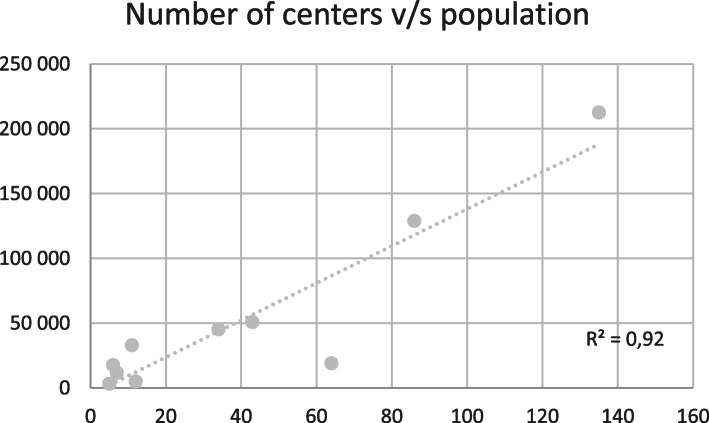
Number of centers v/s population

Using CEPAL information about countries’ population, education GDP, and health GDP, we graphed the relationship with the number of simulation centers. There is a positive linear correlation between the number of centers v/s population (correlation coefficient 0,922) (Fig. 1)

**Fig. 2:**
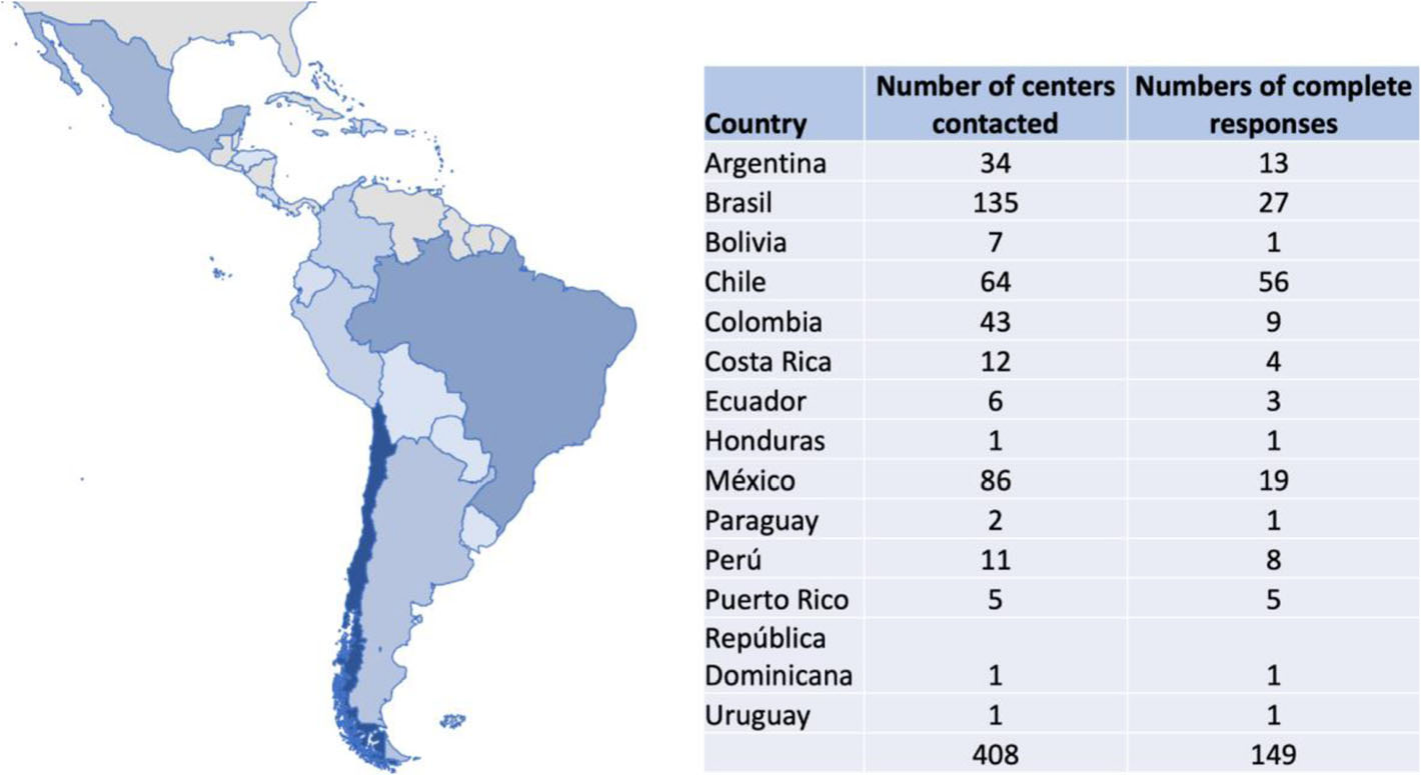
Number of centers contacted by country and complete responses to quality self-perception survey

149 directors sent complete responses (36.5%) on the 42 quality self-perception scale and considered valid on further analyses related to the quality of the programs. Valid responses were obtained from 14 countries. The countries with the highest response rate were Chile (37.5%), Brazil (18.1%), and Mexico (12.7%) (Fig. 2).

Most simulation centers were university linked (84%), and only 12 centers linked to health institutions were reported, of which 50% were located in Chile.

**Fig. 3:**
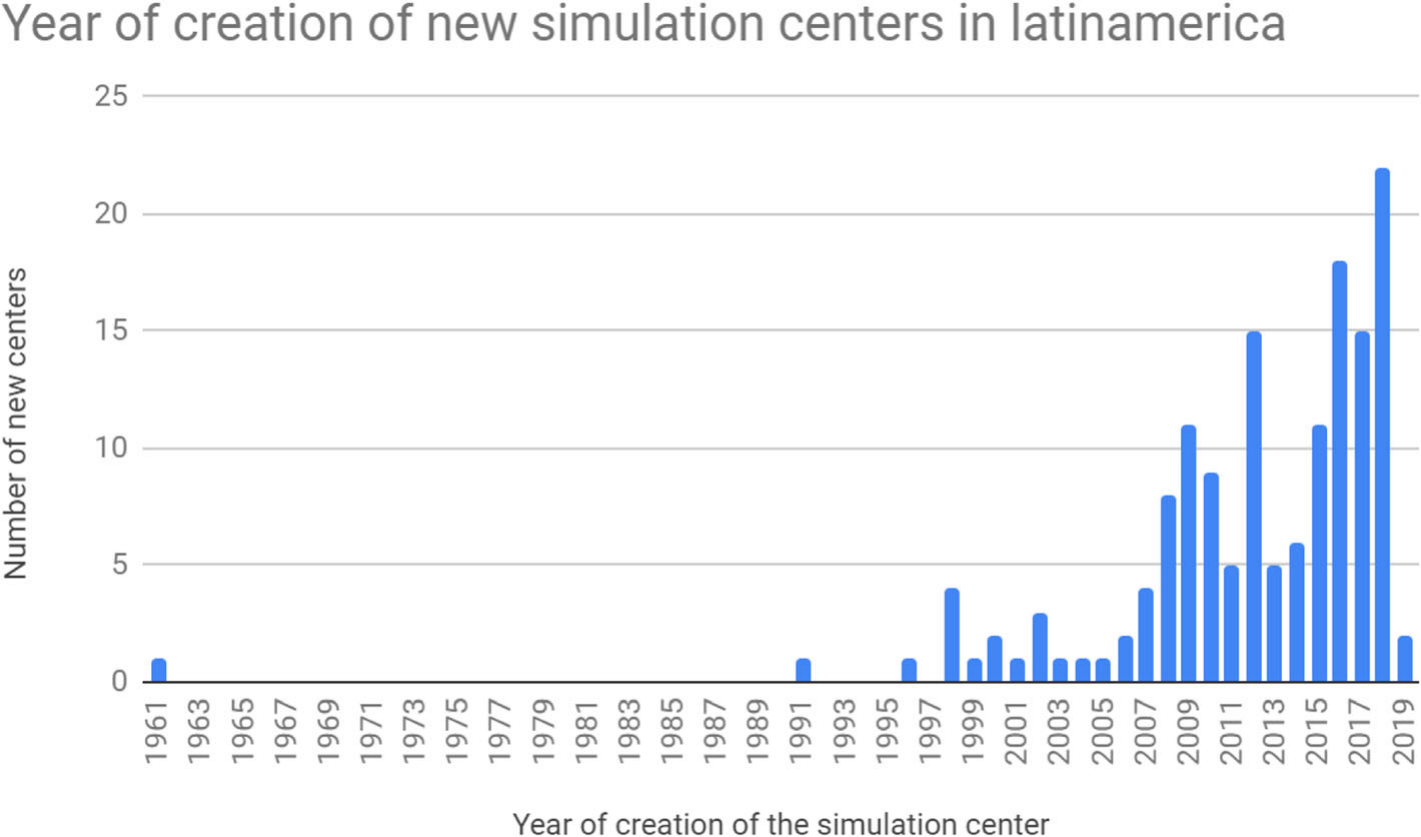
Year of creation of new simulation centers in latinamerica

Centers responding to the survey were in existence for an average of 7 years with a standard deviation of 6833 years and range from 0 to 58 years. The first simulation center in Latin America was created in 1961 in Peru and is still open to date. It is a surgical simulation center that reports that the resources are mainly biological models and surgical trainers. New simulation centers were created every year from 1998 to 2019 (Fig. 3). Between 2008 and 2016, about ten centers per year were created, and in the years 2017 and 2018, we observed the creation of about 20 centers per year. During the first half of 2019, 8 new simulation centers were created in the region.

### Infrastructure and metric of activities

The data reported in the logistics aspects are heterogeneous; the centers’ area is declared between 8 and 4307 m^2^. The information was organized into quartiles, and the centers were categorized into small <122 m^2^, medium 122–1500m^2^, large 1500–2900m^2^, and very large> 2900m_2_. Twenty-five percent of simulation centers are small, and 71% are medium size. Heterogeneity was found in the number of participants per year, hours per participant, number of activities, and number of hours of room use (Table 1).

**Table 1:** Characteristics of Latin American simulation centers

Characteristics	Number of valid responses	Minimum	Maximum	Average	Std. deviation
Center square meters	143	8	4307	425.9	598.5
Number of participants (count the people who participate in each sesion and if someone participates in more than one can count it twice or more. Calculate based on the last full year)	96	6	60000	3116.7	8171.1
Number of hours per participant (1 h per participant= 1 h class x 1 participant. If a class lasts 4 h and had 10 participants, that is 40 h per participant. Calculate based on the last full year)	69	2	3672227	59,711.4	441,908.7
Number of activities (each activity refers to the same block of hours during a day, where a group of participants performed the same activity. Calculate based on the last full year)	73	2	4300	413.6	898.2
Number of hours of room use (multiply the number of rooms by the class time. If there are four rooms used for 4 h of class time, calculate 16 h of room use. Calculate based on the last full year)	71	2	74574	2912.9	9364.3
Director’s age	149	23	75	43.3	13.1
Number of programmatic orientations	149	1	8	4.7	1.7

### Human resources and directors’ profile

Most simulation centers (54%) have less than ten instructors and 8% more than 50. Regarding the profile of the simulation center directors, 61.7% are women, 50% are medical doctors (MD), 40% nurses, and 5% engineers. Thirty-seven percent have a clinical specialty, 53% have a master’s degree, and 13% have doctoral training. In the specific training in simulation, 75% have completed an instructor course, 6% have completed a fellowship in simulation, 5.4% report having Certified Healthcare Simulation Educator (CHSE), and 17% report not having specific training in simulation-based education.

### Simulation resources and programs’ orientation

Regarding the types of simulation resources used in simulation centers, the most commonly used are high-cost simulators (81%) and simulators for procedures (79%). The third place among the simulation resources used in the centers corresponds to simulated patients. The least used resources correspond to biological samples and animal models, which are used in centers dedicated to surgical training (Table 2).

**Table 2:** Distribution of centers who use simulation resources and declare orientation of simulation programs (*n* = 149)

Type of simulation resource used	Number of centers who use that resource (***n***)	Percentage of center who use that resource (%)
High-tech simulators	120	81.1
Task trainers	117	79.1
Simulated patients	87	58.8
Standardized patients	84	56.8
Moulage	75	50.7
Handmade simulators	46	31.1
Virtual simulators	46	31.1
Bench surgical simulators	33	22.3
Biological samples for surgery	27	18.2
Animals for surgery	10	6.8
**Orientations of the simulation programs**	**Number of centers who declare orientations of the simulation programs (*n*)**	**Percentage of centers who declare orientations of the simulation programs (%)**
Teaching and evaluating clinical and procedural skills	140	94.6
Promote critical thinking and problem-solving skills	138	93.2
Promote communication and teamwork	125	84.5
Develop patient safety	110	74.3
Introduce and promote interprofessional learning and practice	84	56.8
Develop care that considers cultural variables	41	27.7
Explore and analyze health systems practices	41	27.7
Specific training to present or take entrance exams to medical-surgical residency	24	16.2

Regarding the intention of acquiring new simulators for next year, 65% of respondents do not want to.

When asked about program orientations, most centers report that their programs intend to improve the practice and development of clinical skills (94%), critical thinking (93%), and human factors (84%) (Table 2).

### Self-perception of quality

The research dimension was the one with the lowest Likert average (*m* = 3.3), and the SSH teaching/learning dimension the one with the highest Likert (*m* = 4.1) global score by dimensions (Table 3).

**Table 3:** Mean average of Likert points by every item of quality self-perception instrument (ASPIRE and SSH criteria) (*n* = 149)

Items	Likert Average
**Aspire-based criteria**	**3.8**
1. Your simulation center has clear objectives aligned with the priorities and objectives of your institution and/or manages to influence the culture of your organization.	**4.2**
2. Your simulation center has a policy or definitions to guarantee that the development of its programs is carried out using a systematic approach to curriculum design, considering learning theories that support its programs.	**4.1**
3. Your simulation center has a policy or definitions to evaluate the implemented programs and thus promote continuous improvement in its practices.	**3.8**
4. At your simulation center, a systematic process is used to align simulation technologies and methodologies with your defined training needs.	**3.8**
5. In your simulation center, the development of educational programs is carried out using the evidence of simulation effectiveness for teaching and training as a guide.	**4.0**
6. In your simulation center, a rigorous and standardized process is used to develop and implement validated performance evaluation instruments (considering their use for training and summative purposes).	**3.7**
7. Your simulation center incorporates evidence-based feedback and debriefing methods for training purposes.	**4.3**
8. Your simulation center uses a continuous and systematic process of quality assurance and continuous improvement of its simulation programs.	**3.9**
9. Your simulation center expects its teaching, administrative and technical staff to have experience in simulation-based education and supports its development by providing the resources necessary to achieve its objectives and maintain its activities.	**4.1**
10. The institution’s simulation program has faculty experienced in conducting simulation-based educational research and supports its development by providing the resources necessary to achieve its objectives and maintain its activity.	**3.3**
11. At your center, innovation in simulation-based education is promoted.	**4.0**
12. The faculty of the institution (or its students) conducts research related to simulation-based education.	**3.0**
13. The centre’s teachers promote simulation-based education nationally and internationally.	**3.5**
**Core criteria SSH**	**3.8**
14. There is a clear and publicly stated mission that specifically addresses the intent and functions of the simulation program, and how the program is linked to the larger organization, if it exists.	**3.8**
15. There is an organizational framework that provides adequate resources (fiscal, human and material) to support the mission of the program.	**3.9**
16. There is a strategic plan designed to accomplish the mission of the program.	**3.7**
17. There are written policies and procedures to ensure that the program provides high quality services, and that it meets its obligations and commitments.	**3.7**
18. The program has a process to determine which simulation modalities and relevant technologies are selected for use in various education, evaluation, research and / or systems improvement activities.	**3.5**
19. The program has a method of evaluating its general areas of programs and services, as well as educational, evaluation, and / or research activities so that they provide feedback for continuous improvement.	**3.4**
20. All activities, communications and relationships demonstrate a commitment to the highest ethical standards.	**4.4**
21. Adequate documentation and organizational policies and mechanisms are in place to ensure that data / evidence security and student confidentiality are maintained.	**3.9**
22. The program demonstrates a commitment to advocating for health simulation and contributes to the field of simulation.	**4.2**
**Teaching/learning criteria SSH**	**4.1**
23. The program offers comprehensive learning activities using simulation.	**4.3**
24. The program provides expert guidance for simulation education for instructors / educators and students.	**3.8**
25. Educational methods are reliable, valid, attractive and, when possible, based on evidence.	**4.1**
26. Appropriate simulation modalities are used to support learning objectives and design.	**4.2**
27. There is access to qualified educators for the educational activities provided. *	**4.2**
28. Curriculum design follows a rational process based on the theory of education currently understood.	**4.0**
29. Simulation activities are carried out in a suitable environment to optimize the achievement of learning objectives.	**4.4**
30. The program continually updates and improves its courses.	**4.1**
31. The program has a demonstrated ability to offer continuing education credits.	**3.4**
**Assessment criteria SSH**	**3.8**
32. The facilities, technology and simulation modalities, as well as standardized patients, and equipment are appropriate for the summative assessment of individual and team knowledge and / or skills.	**4.1**
33. There are qualified consultants and staff to carry out the evaluation activities.	**3.9**
34. There is a systematic process for selecting the appropriate assessment tools.	**3.7**
35. There is adequate support for data analysis.	**3.5**
**Research criteria SSH**	**3.3**
36. The mission statement includes a specific and credible commitment to investigative activities.	**3.4**
37. Instructors/educators/researchers demonstrate ability to conduct research.	**3.5**
38. There is a designated individual who is responsible for administering the research programs.	**3.0**
39. The program emphasizes and supports the application of academic approaches to evaluate teaching, evaluation and / or systems integration programs and to carry out validation studies of systems, approaches or simulation modules.	**3.1**
40. Research protocols are in accordance with accepted research standards.	**3.3**
**Systems integration criteria SSH**	**3.5**
41. The program works as an integrated institutional resource for Safety, Quality and Risk Management that uses the principles of Systems Engineering, Human Factors, Quality, Safety, and/or Risk Management and engages in two-way feedback to achieve business-level objectives and improve the quality of care.	**3.4**
42. The program has an established and committed role in the institutional processes of Quality and Safety Assessment.	**3.5**

The individual descriptors in which the Likert average was higher were, in decreasing order, those related to adequate learning environments (item 29, SSH Teaching/Learning Criteria, *m* = 4.4) ethical aspects (item 20, SSH CORE Criteria, *m* = 4.4) and reflective training techniques (item 7, ASPIRE Criteria, *m* = 4.3) as seen in Table 3.

The lowest average Likert in individual descriptors was 3.0 in item 12, corresponding to ASPIRE Criteria: “The faculty of the institution (or its students) conducts research related to simulation-based education,” and in item 38, corresponding to SSH Research Criteria: “There is a designated individual who is responsible for administering research programs” (Table 3).

The higher score by dimension was obtained using ASPIRE criteria, and the lowest in SSH Research dimension (Table 4).

**Table 4:** Total Score (Sum of total scores of quality self perception instrument) and Total Dimension Score (Sum of Scores by each dimension of quality self perception instrument)

	Ideal score	Mean of Mean Scores	Std. Deviation of mean scores	Percentage of mean scores obtained over ideal score
ASPIRE	65	48,6	12,3	74,8%
SSH Core	45	30,7	13,0	68,2%
SSH Teaching/Learning	45	32,2	13,5	71,6%
SSH Evaluation	20	13,5	5,8	67,7%
SSH Research	25	14,5	7,0	58,1%
SSH Systems Integration	10	6,1	3,1	61,3%
Total Scale	210	145,7	38,3	69,4%

## Discussion

Few studies show the specific characteristics of the simulation centers [18, 19], but they do not consider the orientations of the programs carried out in them. Our work is the first on a large scale in Latin America, and we found an acceptable response rate compared to works in a single country in the region [14].

In the recent Italian survey of simulated pediatric training, nearly 15% of the surveyees answered [21]; In Switzerland, Stocker obtained a response in 96% of hospital centers where they offered training in pediatrics; of these, 66.6% used clinical simulation in their teaching practice [22]. Sixty-six percent of residents surveyed, and 100% of program managers responded to the Canadian emergency medicine training survey [20].

Our response rate is lower than that reported in other latitudes and contexts. It may be given by the cultural diversity in Latin America, by the differences in the development of the simulation in the different countries of the region, or because this is the first time that is carried out surveys of this type.

The number of simulation centers in Latinamerican Countries shows large differences. Since these are countries with different economic and population sizes, it is convenient to compare not only in absolute terms, but also in relation to the level of wealth and number of inhabitants of the countries. We found a positive linear correlation between the number of centers v/s population, with a country that deviates from the tendency, with a higher proportion of centers for the number of inhabitants. Moreover, it is precisely this country that reports the most simulation centers associated with clinical institutions, an area in which it is possible that the rest of Latin America will expand simulation centers in the future given the worldwide trends on systems integration of clinical simulation [20–22]. We do not believe we can recommend a standard of centers per number of inhabitants at the present time or estimate how much this growth will reach a plateau.

We cannot find correlation between global GDP in education or GDP in health and number of simulation centers. One particularity of latinamerican countries are the differences about education funding. In some countries, the expenditure of education depends mostly on private funding, contrasting with the rest of Latin American countries [17].

The size and activities of the Latin American simulation centers were heterogeneous. This may be explained by the fact that some universities have several campuses in different regions of the same country, and the report shows the total activities as a single center, due to their administrative organization of the programs. Another explanation is that some centers conduct a significant number of OSCE evaluations to their own students and in processes of re-validation of international professional degrees, which influences the reporting of higher indicators or that some centers are dedicated to single professions and others attend multiple careers with large groups of students.

At the time of data collection, most of the simulation centers in Latin America were linked to university institutions. It is important to consider that centers linked to clinical institutions may have different forms of organization and focuses of action than those of university institutions, and that the number of centers or simulation activities with a focus on clinical teams may be modified given the need for training of specific clinical competencies related to the current pandemic context.

It was reported that a simulation center using surgical simulation resources was created in 1961. Given the methodology defined in this study, based on self-reporting, no actions were taken to verify this statement. The literature describes the use of frozen biological material for surgical procedural training in 1986 [29], and in the early 1990s, the first recommendations for surgical training with simulation are found [30].

In our work, the professional profile of those who run the centers is heterogeneous in the profession, clinical specialty, academic degrees, and simulation training. The vast majority have received instruction with short courses, and only a few have fellow or international certifications. It is noteworthy that almost one sixth of them do not have specific training in the area.

Observing quality criteria is necessary for human activities that aim for excellence; this includes clinical simulation. In this study, the quality standards used were recognized as highly relevant by the directors of the simulation centers, mainly in teaching, learning environments, and ethical criteria. The research criteria were considered less relevant; this is consistent with the low region research visibility in the world ranking in the last decade [31].

Most of the simulation centers in the region were reported to be linked to universities. This may be related to the fact that the best rated dimensions globally correspond to those based on the ASPIRE criteria and the SSH teaching and learning criteria.

In 2013, Arthur et al. conducted a study with Delphi methodology in which the importance of maintaining standards in nursing simulation-based education is denoted [32]. Although the daily activity of the Latin America centers (participants, activities, etc.) was heterogeneous and relatively low, most of them showed a high number of activities related to the modalities of simulation resources used.

This study presents some limitations, such as the dependence of the self-report and the sincerity of the respondents [33], and the majority of responses come from three countries.

Another important consideration is that snowball sampling makes it challenging to determine the sampling error or make inferences about populations based on the obtained sample.

However, the internal consistency of the survey was high, and the responses were similar by country, size of the center, and profile of the director, which gives greater validity to the results [24]. Another research that attempts to characterize centers worldwide has a lesser response rate than our research [18].

In this case, the information that we get is important because it is the first attempt to characterize the complete region. We consider that this work is a basis to better understand how simulation centers operate in Latin America and open the opportunity for new research in this field.

Some of these research areas are the differences between simulation centers based in university and clinical institutions, or the relevance of the training of center directors in the development of educational programs. It is also important to inquire into the potential differences between self-reporting and independent observer evaluation.

## Conclusions

Simulation-based education in health sciences has had an increasing development in Latin America. Growth alone is not a goal, and quality might be worth looking at.

Characterized centers are predominantly medium-sized, university-based, using standardized mannequins and patients to train clinical skills and procedures.

Agreement with the importance of quality and continuous improvement is high; it is low concerning research criteria and adherence to program evaluation mechanisms.

We recommend starting accreditation processes in Latin America and studies that measure the quality of simulation-based education in our region, based on objective observations more than in self-reporting.

## Data Availability

The datasets used and/or analyzed during the current study are available from the corresponding author on reasonable request.

## Abbreviations

OSCE: Objective and Structured Clinical Examination
SSH: Society for Simulation in Healthcare
AMEE: Association for Medical Education in Europe
INACSL: International Nursing Association for Clinical Simulation and Learning ASPE Association of Standardized Patient Educators
FLASIC: Federación Latinoamericana de Simulación Clínica - Latin American Federation of Clinical Simulation
CEPAL: Confederación
